# Pomalidomide-induced changes in the pancreatic tumor microenvironment and potential for therapy

**DOI:** 10.18632/oncoscience.486

**Published:** 2019-08-23

**Authors:** Peter Storz

**Affiliations:** Mayo Clinic, Griffin Building, Jacksonville, FL, USA

**Keywords:** Pancreatic Cancer, Macrophages, Pomalidomide

In pancreatic cancer, standard chemotherapy alone or its combination of with checkpoint inhibitors is largely ineffective, because the tumor microenvironment generates a fibrotic barrier for immunotherapy and for drugs to reach tumor cells. Current most promising efforts are strategies that combine chemotherapy with compounds that alter the tumor microenvironment. Here we discuss treatment with pomalidomide as a method to target immunosuppressive alternatively-activated tumor-associated macrophages, resulting in a decrease in fibrosis and formation of an immune-responsive environment.

The pancreatic tumor microenvironment (TME) is an immunosuppressive, fibrotic barrier. It blocks the delivery of drugs that target tumor cells, but also excludes immune cells and prevents immunotherapy [1]. Major cell types in the TME are different populations of activated fibroblasts, and immune cells, including tumor-associated macrophages (TAMs). Alternatively-activated (M2) macrophages represent approximately 85% of TAMs in the pancreatic tumor microenvironment [2]. In pancreatic ductal adenocarcinoma (PDA) these macrophages regulate two hallmarks of immune escape, the exclusion of cytotoxic T lymphocytes and fibrosis [3, 4]. Both, either targeting immunosuppressive alternatively-activated TAMs, or their repolarization to inflammatory macrophages, which drive destruction of the tumor stroma and presence of cytotoxic T cells, could be efficient strategies for this cancer [3-5].

Indeed, preclinical data indicate that neutralization of IL-13, a factor that mediates M2 polarization of macrophages, decreases the presence of alternatively-activated macrophages, as well as fibrosis at pancreatic lesions [4]. In recent work, Bastea *et al*. now show that pomalidomide, a thalidomide analog that has been developed and tested for hematologic cancers [6], not only induces a decrease in alternatively-activated macrophages, which then results in decreased fibrosis at PanIN lesions and tumors, it also reprograms these populations into tumor suppressive macrophages [7].

Effects of pomalidomide on M2 macrophages are due to downregulation of interferon regulatory factor 4 (IRF4), a transcription factor for M2 macrophage polarization. Through its effects on macrophage populations pomalidomide generates a pro-inflammatory environment by decreasing tissue levels of interleukin 1 receptor antagonist (IL-1ra) and increasing Interleukin 1α (IL-1α), with the net effect of activating interleukin 1 receptor (IL-1R) signaling [7]. It had been shown previously that pancreatic tumors deficient of IL-1α have an immunosuppressive environment due to exclusion of cytotoxic T cells [8]. As expected, due to re-establishing IL-1R signaling, pomalidomide induced presence of activated (IFNγ-positive) CD4+ and CD8+ T cell populations [7]. This is in line with studies showing that in the pancreas shifting M2 to M1 populations orchestrates effective T cell immunotherapy [9]. In addition to its effects on immune cell populations, combination of pomalidomide with standard of care chemotherapy, recently had been shown to promote chemosensitization [10].

Above preclinical data, and the fact that pomalidomide and other thalidomide analogs are already FDA-approved drugs, makes them ideal candidates for clinical trials focusing on combination therapy with standard of care drugs or immunotherapy. A recently completed phase I clinical study showed that combination of pomalidomide with gemcitabine is feasible and safe for patients with untreated advanced carcinoma of the pancreas [11]. Potential side effects for human use of pomalidomide are minimal as only 2-4% of patients observed treatment-induced adverse events, which can be easily prevented by additional administration of an anticoagulant or aspirin. Pomalidomide/Gemcitabine therapy may be even more efficient when combined with other clinical approaches to target TAMs and immunosuppressive monocytes and sensitize pancreatic tumors to T cell immunotherapy such as inhibition of focal adhesion kinase, anti-PD1 therapy, of CD40 agonists, or targeting of CCL2 (reviewed in [1]).

**Figure 1:**
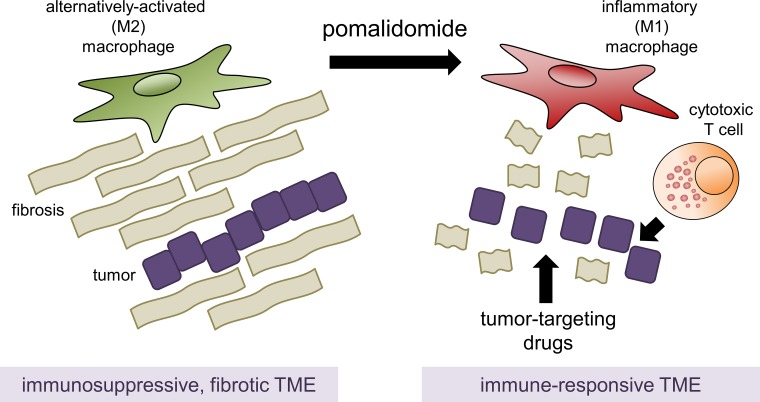
Pomalidomide-mediated changes in the pancreatic tumor microenvironment and potential for therapy M2 polarized, alternatively-activated TAMs drive fibrosis and generate an immunosuppressive environment, preventing efficient therapy. Pomalidomide induces a polarization switch from M2 to inflammatory M1 macrophages, and also drives the recruitment of activated cytotoxic T cells into the pancreatic TME. This leads to an immune-responsive TME, showing a decrease in fibrosis, but also and makes tumor cells more accessible for standard of care chemotherapy.

In summary the data of Bastea *et al*. [7] suggest that pomalidomide holds promise for pancreatic cancer therapy, by remodeling the tumor microenvironment and generating a shift from an immuno-suppressive to an immune-responsive environment (Figure 1).
